# Disulfiram in glioma: Literature review of drug repurposing

**DOI:** 10.3389/fphar.2022.933655

**Published:** 2022-08-24

**Authors:** Shiyu Zhong, Shengyu Liu, Xin Shi, Xudong Zhang, Kunhang Li, Guojun Liu, Lishuai Li, Shanwei Tao, Bowen Zheng, Weichen Sheng, Ziyin Ye, Qichen Xing, Qingqing Zhai, Lijie Ren, Ying Wu, Yijun Bao

**Affiliations:** ^1^ Department of Neurosurgery, The Fourth Hospital of China Medical University, Huanggu, Shenyang, China; ^2^ School of Maths and Information Science, Shandong Technology and Business University, Yantai, China; ^3^ Business School, All Saints Campus, Manchester Metropolitan University, Manchester, United Kingdom; ^4^ Luoyang Central Hospital Affiliated to Zhengzhou University, Luoyang, China; ^5^ School of Management, Shanghai University, Shanghai, China; ^6^ Neurology Department of Shenzhen Second People’s Hospital/ First Affiliated Hospital of Shenzhen, University Health Science Center, Shenzhen, China; ^7^ Department of General Practice, The First Hospital, China Medical University, Shenyang, China

**Keywords:** gliomas, glioblastoma multiforme, disulfiram, drug repurposing, temozolomide

## Abstract

Gliomas are the most common malignant brain tumors. High-grade gliomas, represented by glioblastoma multiforme (GBM), have a poor prognosis and are prone to recurrence. The standard treatment strategy is tumor removal combined with radiotherapy and chemotherapy, such as temozolomide (TMZ). However, even after conventional treatment, they still have a high recurrence rate, resulting in an increasing demand for effective anti-glioma drugs. Drug repurposing is a method of reusing drugs that have already been widely approved for new indication. It has the advantages of reduced research cost, safety, and increased efficiency. Disulfiram (DSF), originally approved for alcohol dependence, has been repurposed for adjuvant chemotherapy in glioma. This article reviews the drug repurposing method and the progress of research on disulfiram reuse for glioma treatment.

## 1 Introduction

Glioma is a central nervous system (CNS) tumor and is one of the most common malignant brain tumors (Özcan et al., 2021), accounting for approximately 80% of all brain-related malignancies (Ostrom et al., 2019). According to the World Health Organization (WHO) classification, gliomas are divided into four grades based on their malignancy. Grades I–II are low-grade gliomas (LGGs), whereas grades III–IV are called high-grade gliomas (HGGs) (Özcan et al., 2021). Glioblastomas (GBMs) are WHO grade IV tumors with a high degree of malignancy and a median overall survival of approximately 15–26 months (Seliger and Hau, 2018).

Conventional treatment only modestly prolongs survival (Jakola et al., 2018). The growing demand for effective anticancer drugs has led researchers to search for Food and Drug Administration (FDA)-approved drugs that can be reused as chemotherapeutic agents (McMahon et al., 2020). Drug repurposing, also known as drug repositioning, drug reprofiling, drug redirecting, drug rediscovery, and others (Langedijk et al., 2015), is a promising treatment strategy for reusing drugs with known formulations, pharmacokinetics, toxicity, clinical trials, and post-marketing surveillance safety data that offer increased scope for use (Ashburn and Thor, 2004; Turanli et al., 2021).

**FIGURE 1:**
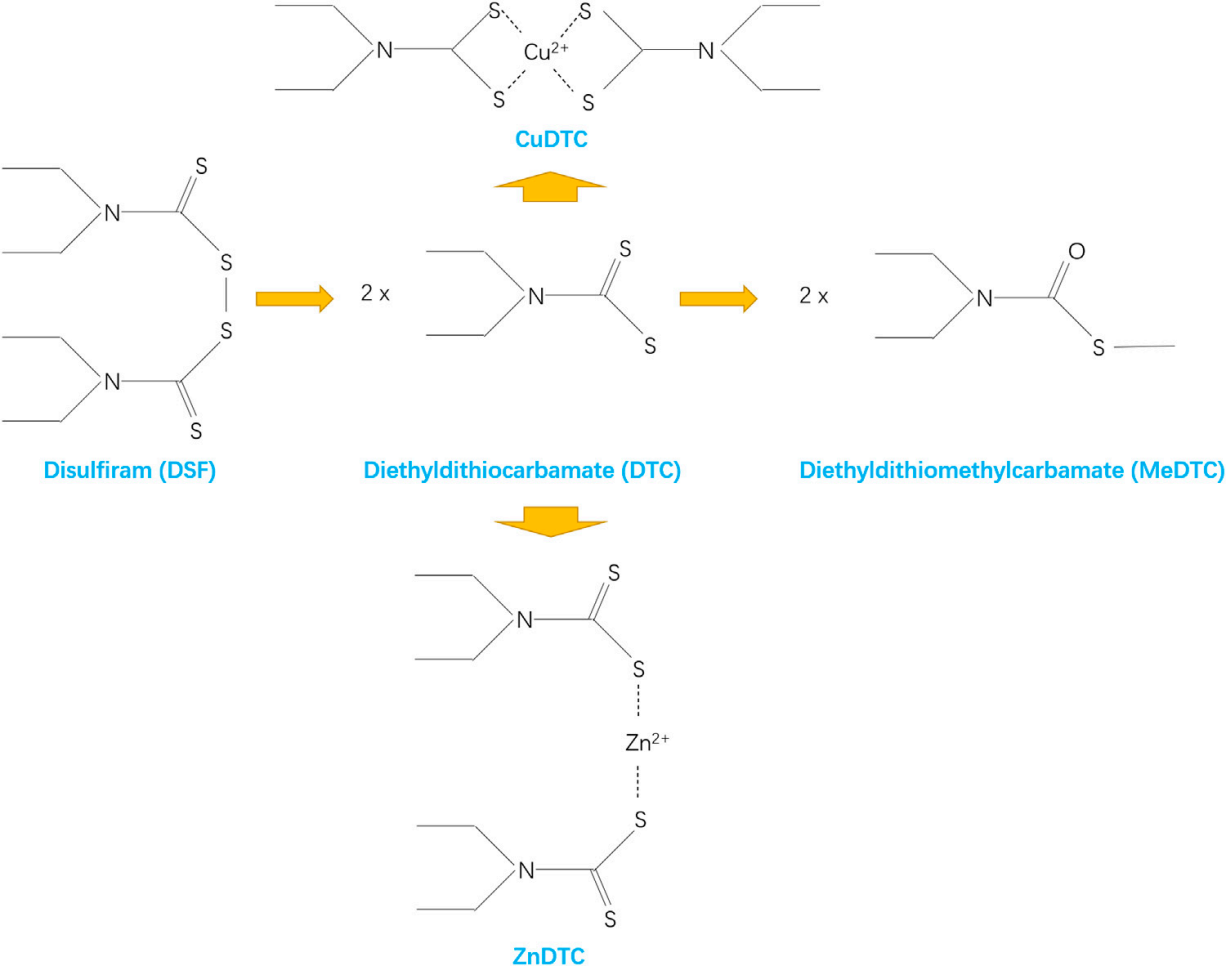
Molecular structure of DSF.

Disulfiram (DSF), also known as antabuse, is an FDA-approved acetaldehyde dehydrogenase inhibitor (Shirley et al., 2021; Turanli et al., 2021). Figure 1 shows the molecular structure of DSF. It has been used for treating alcohol dependence for the past 70 years with good pharmacokinetic properties, safety, and tolerability owing to the flu-like symptoms that patients experience when consuming alcohol (Seliger and Hau, 2018; Shirley et al., 2021). Preclinical and clinical studies have shown that DSF exhibits broad-spectrum anticancer activity against a variety of cancer types when administered with copper (Cu)-containing supplements (McMahon et al., 2020). Therefore, this review describes the drug repurposing of DSF in gliomas.

## 2 Chemotherapy for glioma

The standard initial treatment for GBM is extensive neurosurgical resection, followed by postoperative segmental radiotherapy, temozolomide (TMZ) chemotherapy, and combined adjuvant therapy (Jakola et al., 2018; Stylli, 2020). The therapy using some natural and synthetic anti-glioma agents, such as medicinal cannabis or cannabinoids, bipolaris setariae fungi, oncolytic viruses, neurostatin, and fatty acid synthase (FAS) inhibitors, can also help combat gliomas (Anjum et al., 2017). Despite comprehensive strategies, residual GBM cells can develop treatment resistance (TR), resulting in GBM recurrence within a median time of approximately 7 months (Louis et al., 2016; Liu C. C. et al., 2021).

TMZ is a chemotherapeutic agent specifically used for brain cancer and was approved for recurrent mesenchymal astrocytoma in the United States in 1999 and recurrent mesenchymal astrocytoma and glioblastoma in Europe in 2000 (Mutter and Stupp, 2006). However, mitozolomide, the essential compound of TMZ, entered the clinic in 1983 (Newlands et al., 1997) and a phase I trial was completed in 1985 (Newlands et al., 1985), indicating that TMZ was developed over 16 years. A randomized clinical study conducted by Stupp in 2005 showed that adding TMZ to radiotherapy improved the 2-year survival rate of patients with GBM from 10.9% to 27.2% with minimal toxicity (Stupp et al., 2005). Since then, TMZ has been used as the first-line chemotherapeutic agent for gliomas (Stylli, 2020).

However, it is estimated that at least 50% of patients treated with TMZ do not respond to it, exhibiting innate or acquired chemoresistance, which ultimately leads to tumor recurrence (Lee, 2016). Presumably, during the window of chemotherapy with TMZ, most tumor cells are vulnerable and killed and may not have acquired full resistance (Osuka and Van Meir, 2017). Eventually, tumor cells resistant to multiple therapies persist in the brain parenchyma surrounding the tumor cavity and become the basis of tumor regeneration and recurrence (Osuka and Van Meir, 2017).

Given the slow and costly development of new therapies, drug repurposing has become an attractive strategy (Stylli, 2020). For example, a trial on metformin for GBM in 2015 showed that metformin prolonged the survival of patients with glioblastoma and diabetes (Adeberg et al., 2015). However, a pooled analysis questioned this finding and showed that metformin did not prolong survival in patients with GBM (Seliger et al., 2020). Similarly, celecoxib, as an adjuvant to TMZ chemotherapy, has shown good tolerability, but its efficacy in terms of survival benefits for patients remains uncertain (Stockhammer et al., 2010). The classical antimalarial drug, chloroquine, has been found to kill various cancer types, including GBM, through drug repurposing (Weyerhäuser et al., 2018). However, its concentration threshold for killing tumor cells under laboratory conditions is much higher than the clinically tolerable dose; therefore, its clinical value remains unclear (Weyerhäuser et al., 2018).

TMZ has shown great therapeutic value; however, the rate of drug resistance and recurrence of glioma remains reasonably high. Drug repurposing has contributed to the discovery of new therapies, but clinical application remains slow. Thus, there is an urgent need for developing new adjuvant chemotherapeutic agents.

## 3 Drug repurposing

### 3.1 Significance of drug repurposing

Drug repurposing can shorten development times by 5–7 years, reduce research investment costs by accounting for less than 10% of new compounds, and avoid risks such as a constant weight of generics (Cha et al., 2018). Moreover, a strict and burdensome regulatory process for new drugs forces developers to explore novel therapeutic uses for existing drugs (Turanli et al., 2021). Drug repurposing provides sufficient assurance regarding the safety, efficacy, and administration route of existing drugs (Parvathaneni et al., 2019; Juárez-López and Schcolnik-Cabrera, 2021; Turanli et al., 2021). Studies have shown that the cost and time to develop DSF as an anticancer drug are reduced by over 40% with an estimated annual cost of approximately $550 with DSF 500 mg per day (Cvek, 2012).

Drug repurposing facilitates the passage of projects and improves development efficiency. Only a minority of drug development projects can obtain FDA approval among newly developed drugs, compared with over 65% of drug repurposing projects (Masoudi-Sobhanzadeh et al., 2019). Recently, drug repurposing projects have accounted for approximately 30% of all newly approved drugs by the FDA (Parvathaneni et al., 2019). Additionally, drug repurposing can allow the revaluation of drugs that failed in the development phase for other uses and the change of application settings for better use (Masoudi-Sobhanzadeh et al., 2019).

### 3.2 Methods of drug repurposing

As high-throughput screening and computational biology methods advance, accumulated data lay the foundation for new approaches to rational drug repurposing (Turanli et al., 2021). Currently, numerous drug repurposing databases are available, allowing easy access to drug repurposing research, and systematic analysis is now accessible on platforms or screening systems dedicated to identifying repurposable drug candidates (Juárez-López and Schcolnik-Cabrera, 2021).

There are multiple information-gathering methods for drug repurposing. Electronic drug repurposing involves using various public databases to gather information from research, clinical trials, utilization reports, and other published data, and then to identify drug targets and networks of drug-drug interactions with the help of bioinformatics tools and artificial intelligence (Kumar et al., 2019). Text mining methods are used to discover new information by extracting aggregated new information from multiple published resources with the help of a computer, and by obtaining a large amount of data on conceptual relationships in biology from publications (Kumar et al., 2019). Clustering methods are applied to display and discover new drug targets or drug-disease relationships through various modules, groups, or subnetworks using clustering algorithms (Kumar et al., 2019). Propagation methods are based on information transmission from the source node to the network nodes and individual subnetwork nodes to determine the relationship between disease genes and target diseases (Emig et al., 2013). Semantic approaches seek biological entity relationships from medical databases, build semantic networks based on existing ontology networks, develop algorithms to discover the relationships, and extract medical information and image resources for drug reuse (Xue et al., 2018). Biological approaches include using systems and network biology to develop various models for drug reutilization studies that mimic the physiological environment of the target protein and modulate the outcome of its action, particularly targeting multifactorial complex diseases (Pujol et al., 2010). Knowledge-based empirical approaches are methods based on the knowledge of researchers and physicians and their ability, experience, and skills to interpret observations, with the opportunity for the serendipitous discovery of new drug utilization pathways (Kumar et al., 2019). Experimental methods include target screening, cellular analysis, animal models, and clinical trials (Kumar et al., 2019). These various methods of drug repurposing have been developed to evaluate the clinical efficacy for glioma treatment.

### 3.3 Database for drug repurposing

Advances in biotechnology, bioinformatics, and histological techniques (proteomics, genomics, metabolomics, etc.) have facilitated the development of several databases in biology, chemistry, medicine, and pharmacology (Kumar et al., 2019).

Basic databases for drug repurposing, such as DrugBank (Wishart et al., 2018), DGIdb (Cotto et al., 2018), and KEGG (Kanehisa et al., 2017), can provide information on drugs, targets, and pathways (Masoudi-Sobhanzadeh et al., 2019; Turanli et al., 2021). Recently, the newly developed DrugR + database based on DrugBank and KEGG provides a new source of information for the single application and reuse of drugs (Masoudi-Sobhanzadeh et al., 2019). The DrugR + database supports not only specialist users with structured query language (SQL) query functions, but also nonspecialist users with different options for targeted functions (Masoudi-Sobhanzadeh et al., 2019). The DrugR + database can also be readjusted to include drug use and provide a list of potential drugs for certain uses (Masoudi-Sobhanzadeh et al., 2019). The DrugR + database has several advantages, such as 1) providing a suitable and simple way to search for and obtain information about drugs with no technical problems, 2) providing information on the reusability of drugs, 3) allowing the selection of different types of research targets to obtain lists of drugs for diseases, and 4) providing up-to-date information on new drugs and the latest research (Masoudi-Sobhanzadeh et al., 2019).

Drug repurposing databases contain news, articles, and results obtained from drug repurposing studies (Masoudi-Sobhanzadeh et al., 2019), such as RepoDB (Brown and Patel, 2017b), Excelra (Arora et al., 2017), Drug Repurposing Hub (DRH) (Corsello et al., 2017), and TTD (Yang et al., 2016). Databases such as PREDICT (Gottlieb et al., 2011) and RepurposeDB (Shameer et al., 2018) summarize similarities between one drug or target and another (Masoudi-Sobhanzadeh et al., 2019). ChemMapper and iDrug Target are databases that assess ligand similarity (Sam and Athri, 2019). Drug target-based databases, such as DMAP (Huang et al., 2015), DrugSig (Wu et al., 2017), DDW (Holland, 2016), PDID, and idTarget (Sam and Athri, 2019), summarize drugs and their various targets, based on which new drug application pathways is proposed (Masoudi-Sobhanzadeh et al., 2019).

There are other databases such as ConnectivityMap (Lamb et al., 2006) for drug-induced gene expression studies, LINCS (Koleti et al., 2018) and GEO (Barrett et al., 2013) for transcriptomic characterization of tumor tissue from patients with cancer, CSNAP (Gaulton et al., 2012) and STITCH (Kuhn et al., 2012) for biological networks, RE:fine drugs (Moosavinasab et al., 2016) and MeSHDD (Brown and Patel, 2017a) linking drugs to disease databases, and PubChem (Wang et al., 2017) for comprehensive chemical and structural information on active ingredient components.

These databases cover an amount of information on drug targets, pathways, characteristics, and recent advances, allowing for exploring potentially efficient drugs.

## 4 History of drug repurposing of disulfiram

The urgent need for glioma treatment and the application of DSF to cancer through drug repurposing has led to a steady stream of studies in recent years that have shown good results in treating glioma (Huang J. et al., 2016; Huang et al., 2018; Huang et al., 2019; Halatsch et al., 2021). Table 1 shows the information on DSF in the drug repurposing database.

**TABLE 1:** The information on DSF in the drug repurposing database.

Drug repurposing database	Website	Id number	Disease area	Drug repurposing area
RepoDB	http://apps.chiragjpgroup.org/repoDB/	None	Alcoholic Intoxication, Chronic (CUI: C0001973)	GBM/glioma
DRH	www.broadinstitute.org/repurposing	BRD-K32744045	Abstinence from alcohol (neurology/psychiatry)	None
DrugBank	www.drugbank.ca	DB00822	Chronic Alcoholism	GBM/glioma
DGIdb	www.dgidb.org	NSC-25953	Alcohol Deterrents	Cocaine abuse
KEGG	http://www.kegg.jp	D00131	Management of selected chronic alcohol patients	Antiparasitic
LINCS	http://lincsportal.ccs.miami.edu/	LSM-5467	Alcohol dependence	Melanoma
DrugSig	http://biotechlab.fudan.edu.cn/database/drugsig/	BCTD00137	Chronic alcoholism	None
PubChem	https://pubchem.ncbi.nlm.nih.gov	3117	Alcoholism	GBM

In 1937, factory workers who were regularly exposed to DSF developed flu-like symptoms when they ingested alcohol (Triscott et al., 2015). DSF has been used to treat alcohol dependence since 1947 (Chick, 1999).

In the last 40 years, the anticancer effects of DSF have been discovered *in vitro* and in cancer xenografts (Conticello et al., 2012; Paranjpe et al., 2014). In 2003, Wang et al. suggested the possible clinical use of DSF in rectal cancer using cellular assays (Wang et al., 2003). In 2006, Chen et al. demonstrated through animal testing that DSF promotes selective apoptosis of tumor cells by inhibiting proteasome activity (Chen et al., 2006). In 2009, Iljin et al. systematically investigated the efficacy of most drugs and drug-like molecules already on the market against prostate cancer cells, and ultimately showed that DSF reduced tumor growth, induced metallothionein expression, and reduced DNA replication *in vivo*, indicating its potential as a therapeutic agent for prostate cancer (Iljin et al., 2009).

In 2009, Richard et al. speculated that DSF should be studied as an adjuvant to chemotherapy for glioblastoma based on its effect on acetaldehyde dehydrogenase (ALDH) in glioma (Kast and Belda-Iniesta, 2009), and since then the value of DSF in glioma has been focused. In 2012, Joanna et al. used the database approach for drug repurposing and selected DSF from numerous drugs that inhibit tumor-initiating cells using the Prestwick database (Triscott et al., 2012). They found that DSF inhibited PLK1 expression in GBM cells, suggesting that DSF could be repurposed for the treatment of refractory GBM (Triscott et al., 2012). In 2015, high ALDH1A1 expression was found to be associated with highly aggressive tumor cells and high-grade gliomas (Chen et al., 2006). ALDH1A1 promoted glioma progression, invasion, and proliferation, and led to poor prognosis (Chen et al., 2006). This finding brought the anti-glioma effects of DSF as an ALDH inhibitor back into the spotlight (Chen et al., 2006). In 2016, Huang et al. conducted the first phase I clinical trial using DSF in combination with TMZ for GBM treatment (Huang J. et al., 2016). In 2017, Karamanakos et al. combined DSF with standard treatment modalities to treat a patient with GBM and ultimately improved his prognosis, thereby confirming the clinical value of DSF (Karamanakos et al., 2017). In 2019, DSF was first reported to preferentially enhance radiosensitivity in GBM cells, particularly in radioresistant cells (Koh et al., 2019). In 2021, Meier et al. demonstrated the therapeutic value of DSF as a repurposed drug for treating gliomas in children (Meier et al., 2021). To date, relevant clinical trials are ongoing (NCT03363659, NCT03151772, NCT02715609, etc.).

## 5 Clinical practice for disulfiram in glioma

Huang et al. (2016) conducted a phase I clinical trial. Twelve patients newly diagnosed with supratentorial primary GBM were studied using concomitant adjuvant DSF and TMZ chemotherapy after radiotherapy. Patients were divided into two groups and received 500 mg/day or 1000 mg/day of DSF in combination with TMZ chemotherapy. The results showed that the maximum tolerated dose of DSF was 500 mg/day. Although some associated adverse effects, such as fatigue, delirium, ataxia, dizziness, and peripheral motor/sensory neuropathy, existed, these adverse reactions were self-limiting and resolved within 30 days after DSF discontinuation (Huang J. et al., 2016).

In 2018, Huang et al. updated data from a phase I clinical trial. The study population comprised 18 patients newly diagnosed with GBM after standard radiotherapy. DSF was also administered during the chemotherapy phase of TMZ. Seven patients received DSF at 500 mg/day, five patients received DSF at 1000 mg/day, and six patients received DSF/Cu at 500 mg/day. The results showed that a maximum dose of 500 mg/day of DSF was well tolerated with or without combined Cu, while 1000 mg/day was poorly tolerated. Of the patients receiving 500 mg DSF with a combination of Cu per day, one patient suffered from nausea and diarrhea in the first 30 days, which was relieved after the reduction of DSF to 250 mg per day. Additionally, without the combination of Cu, one patient developed delirium after 1.6 months and one developed motor neuropathy after 2.6 months. All adverse reactions resolved rapidly after dose reduction or DSF discontinuation. Notably, a 40-year-old woman in the study who received 500 mg DSF per day discontinued DSF therapy after 2.6 months of treatment due to motor neuropathy. At 33 months after DSF treatment, the patient survived in good health with no signs of tumor recurrence (Huang et al., 2018).

Based on a previous study, Huang et al. conducted a phase II clinical trial in 2019. Twenty-three patients with recurrent TMZ-resistant GBM were enrolled in the study. DSF (80 mg) and Cu gluconate (1.5 mg) were administered orally thrice daily at approximately 4–8 hourly intervals in conjunction with TMZ chemotherapy. The results showed that 14% of the patients achieved clinical benefit over a stabilization period of over 6 months. The most common adverse effects were nausea and vomiting in 17% of patients, followed by dizziness (Huang et al., 2019).

In 2021, Marc et al. conducted a phase Ib/IIa clinical trial using a CUSP9 treatment regimen in combination with TMZ. Ten patients with GBM were included in this study. CUSP9 was gradually added at increasing doses during uninterrupted TMZ chemotherapy. When all drugs reached the target dose, the drug was maintained until side effects or tumor progression occurred. The results showed that the regimen was safe under clinical, laboratory, and electrocardiographic monitoring and that the side effects were mild and disappeared after discontinuation (Halatsch et al., 2021).

Since 2016, there have been updates on clinical trials using DSF as an adjuvant in the treatment of glioma. Evidence suggests that DSF has great therapeutic potential but with mild side effects; however, clinical studies are still inadequate.

## 6 Pharmacological mechanism of disulfiram

DSF can act as an anticancer agent through various Cu- and zinc-dependent processes, including the inhibition of nuclear factor kappa B (NF-κB), NPL4, and phosphoglycerol dehydrogenase (Spillier et al., 2019). DSF produces oxidative stress by inhibiting NF-κB activation and superoxide dismutase (SOD) and inducing an increased ratio of oxidized glutathione to its reduced form (Tesson et al., 2017). DSF cytotoxicity is dependent on Cu (Ye et al., 2011). Cu plays a key role in redox reactions and triggers the generation of reactive oxygen species (ROS) in human cells (Liu et al., 2012). Many cancer types, including GBM, have significantly higher intracellular Cu levels than normal tissues, and DSF penetrates and chelates Cu intracellularly (Liu et al., 2012). As DSF cytotoxicity appears to be Cu-dependent, high Cu concentrations in cancer cells produce cytotoxicity through oxidative stress generated by the Fenton reaction (production of Cu ions and hydroxyl radicals) or through inhibiting enzymes that bind Cu to peptide bonds, allowing DSF to specifically target cancer cells and preserve normal tissue (Liu et al., 2012; Tesson et al., 2017).

Approximately 80–95% of the orally administered DSF is absorbed and the unabsorbed portion is excreted (Shirley et al., 2021). In the body, DSF is rapidly metabolized to DTC acid and then rapidly formed as diethylthiocarbamic acid methyl ester (diethyldithiomethylcarbamate, MeDTC) or broken down into carbon disulphide and dimethylamine (Shirley et al., 2021). MeDTC inhibits ALDH and is a strong metal chelate that can form complexes with metal ions (Shirley et al., 2021). DSF is also reduced to DTC in the stomach and forms metal-ion complexes in the gastrointestinal tract (Shirley et al., 2021). DTC metal complexes have a relatively long half-life, are widely distributed throughout the body, and penetrate the blood-brain barrier (BBM) (Shirley et al., 2021). This pharmacological mechanism makes it possible for DSF to kill glioma cells specifically. During DSF treatment, alcohol ingestion leads to acetaldehyde accumulation due to ALDH inhibition, causing the DSF-ethanol reaction (Jørgensen et al., 2011). This reaction manifests as tachypnea, tachycardia, facial flushing, nausea, vomiting, hypotension, and even cardiovascular collapse (Jørgensen et al., 2011).

## 7 Molecular mechanism of disulfiram against tumor stem cells

### 7.1 Inhibition of ALDH

DSF has a symmetric structure, and its first metabolic step is reducing the disulfide bond at the center of the molecule to produce two diethyldithiocarbamate (DTC) fractions (Lipsky et al., 2001a). DTC is further converted to its methyl ester and other metabolites (Lipsky et al., 2001a). DTC is a potent ALDH inhibitor, which forms a mixed disulfide bond with the key cysteine near the active site (Lipsky et al., 2001a). DSF significantly alters alcohol metabolism and treats chronic alcohol dependence by irreversibly inhibiting ALDH and causing acetaldehyde accumulation (Jørgensen et al., 2011). ALDH belongs to a family of metabolic enzymes that catalyze the oxidation of aldehydes, a toxic alcohol metabolism product (Lipsky et al., 2001b). ALDH promotes cell survival by protecting DNA from genotoxic damage and providing resistance to a wide range of anticancer drugs (Hothi et al., 2012). Therefore, ALDH inhibition is an effective way to sensitize resistant cell populations to the cytotoxic effects of chemotherapeutic drugs (Hothi et al., 2012). The strong expression of ALDH is a prominent feature of normal and cancer stem cells, including the stem cell subpopulation of glioblastoma (Kast and Belda-Iniesta, 2009). In GBM and other cancers, increased ALDH expression is observed in a small subpopulation of tumor cells with stem cell properties (Rappa et al., 2013). ALDH expression is associated with the anti-apoptotic capacity of glioma stem cells (GSCs) and the protection of DNA against damage by ROS and aldehydes (Kast and Belda-Iniesta, 2009). DSF and its metabolites form mixed disulfide bonds with key cysteines (Cys302) near the active site of ALDH to inactivate it (Paranjpe et al., 2014). With ALDH inhibition, the division of stem cells into non-stem daughter cells is blocked (Kast and Belda-Iniesta, 2009). However, such inhibitory effect of DSF on ALDH does not affect normal neural stem cells or fibroblasts (Choi et al., 2015).

### 7.2 Induced degradation of mixed lineage leukemia

MLL1 and MLL2 are human homologs of the *Drosophila* epigenetic regulator Trithorax (Trx) (Schuettengruber et al., 2017). MLL1 promotes tumor stem cell characteristics, cell growth, and tumorigenicity in adult glioblastomas (Meier et al., 2021). MLL2 mutations are found in 14% of patients with medulloblastoma (Parsons et al., 2011). Studies have shown that DSF can effectively kill both childhood glioma stem cells at low concentrations and glioma cell lines at slightly higher concentrations by inducing MLL degradation (Meier et al., 2021).

### 7.3 Mediation of increased intracellular ROS production

DSF-mediated cytotoxicity is partially due to increased ROS production. Elevated ROS levels are the major mode of DSF-mediated cell death (Kast et al., 2014). DSF/Cu induces ROS in GBM cell lines, which activates the JNK and p38 pathways, and inhibits NF-κB activity (Liu et al., 2012). DSF/Cu eliminates stem cell-like cell populations in GBM cell lines by modulating the Bcl2 family to trigger the intrinsic apoptotic pathway (Liu et al., 2012). DSF rapidly inhibits superoxide dismutase 1 (SOD1) in murine microglia to induce neurotoxic microglia activation (Dimayuga et al., 2007). ROS production by activated microglia directs redox-sensitive inflammatory signaling and initiates neurotoxic inflammation (Dimayuga et al., 2007). The inhibition of activated microglia might lower the neurological side effects of DSF during the treatment.

## 8 Molecular mechanism of disulfiram against glioma cells

### 8.1 Inhibition of MGMT activity

O6-methylguanine-DNA methyltransferase (MGMT) is a DNA repair protein and chemotherapeutic target that is highly expressed in approximately 80% of brain tumors and other cancers (Gerson, 2002). MGMT, as an anti-mutagenic DNA repair protein, can remove mutagenic O6-alkyl groups from guanine and interfere with the cytotoxic effects of alkylating agents to make tumors resistant (Gerson, 2002). The survival of patients with GBM depends on the MGMT promoter methylation status (Koh et al., 2019). Those with methylated MGMT promoter (MGMT meth) had a higher survival rate than those with the wild-type (MGMT wt) (Paranjpe et al., 2014; Koh et al., 2019). Thus, MGMT has become a central determinant of tumor resistance to alkylating agents (Paranjpe et al., 2014). DSF causes MGMT degradation (Srivenugopal et al., 2016) and synergistically inhibits the growth and renewal of TMZ-resistant GBM cells (Triscott et al., 2012). Compared to normal astrocytes, DSF induces a preferential increase in radiosensitivity in GBM cells, causing increased apoptosis and delayed DNA damage repair (Koh et al., 2019). DSF-induced radiosensitization is more pronounced in radioresistant cells, especially drug-resistant GBM cells with wild-type non-methylated MGMT promoters (Koh et al., 2019). In brain tumor cell lines, DSF reduced MGMT activity in a rapid and dose-dependent manner (Paranjpe et al., 2014). Of these, DSF/Cu was approximately five times more potent than DSF in inhibiting MGMT activity in cultured brain tumor cells (Paranjpe et al., 2014). There are no reports about adverse effects on normal neuronal cells caused by this mechanism.

### 8.2 Inhibition of NF-κB

NF-κB promotes disease progression by increasing tumor cell proliferation, inducing the transcription of anti-apoptotic genes and genes involved in the DNA damage response, and promoting angiogenesis (Voorhees and Orlowski, 2006). NF-κB activation is associated with radioresistance, particularly through inducing anti-apoptotic and antioxidant gene expression (Tesson et al., 2017). DSF is an NF-κB inhibitor, which interferes with TGF-β-induced epithelial-mesenchymal transition in cancer (Han et al., 2015). The blockade of NF-κB activation by DSF reduces tumor volume and cell invasion (Westhoff et al., 2013). The aggressiveness of GBM is associated with the secretion and processing of fibronectin by GBM cells via fibrinogen and matrix metallopeptidases (Mettang et al., 2018). GBM causes reduced intercellular interactions by creating this new extracellular matrix (ECM)-based microenvironment, and generating a stress response that triggers NF-κB activation and enhances cell-matrix adhesion of GBM (Mettang et al., 2018). DSF blockade of NF-κB activation inhibits cell-matrix adhesion between GBM and the brain tissue microenvironment, and reduces tumor volume and cell invasion (Mettang et al., 2018). Other drugs that modulate fibrinogen, metallopeptidases, and fibronectin in the microenvironment of glioma cells might synergistically enhance the anti-invasive effects of DSF.

### 8.3 Inhibition of proteasome activation

Proteasome activity promotes tumorigenesis by enhancing tumor cell proliferation, down-regulating apoptosis, and promoting angiogenesis (Voorhees and Orlowski, 2006). Therefore, the ubiquitin-proteasome pathway is an important target for cancer therapy (Voorhees and Orlowski, 2006). DSF inhibits proteasome activation (Voorhees and Orlowski, 2006), leading to the accumulation of misfolded proteins and potentially toxic protein aggregates (Hothi et al., 2012), subsequently inducing tumor cell death (Hothi et al., 2012). The DSF activity depends on the presence of Cu ions, and Cu thiocarbamate complexes act as proteasome inhibitors (Hothi et al., 2012). As a potent proteasome inhibitor, DSF/Cu can functionally impair the DNA repair pathways and enhance the effects of DNA alkylating agents and radiation (Lun et al., 2016). DSF/Cu inhibits the chymotrypsin-like proteasome activity in cultured glioma stem cells (GSC), consistent with the inactivation of the ubiquitin-proteasome pathway and subsequent tumor cell death induction (Hothi et al., 2012).

### 8.4 Downregulation of PLK1 expression

Polo-like kinase 1 (PLK1), which is highly expressed in tumor cells, is a key serine/threonine kinase involved in many important cell cycle functions such as mitotic entry, centrosome maturation, cell cycle progression, and cytoplasmic division (Triscott et al., 2012). As GBM tumors with higher levels of PLK1 expression have a higher incidence and poorer prognosis, PLK1 could be a promising therapeutic target for brain tumors (Triscott et al., 2012). DSF leads to downregulation of cell cycle kinase PLK1 in GBM cells (Triscott et al., 2012).

**FIGURE 2:**
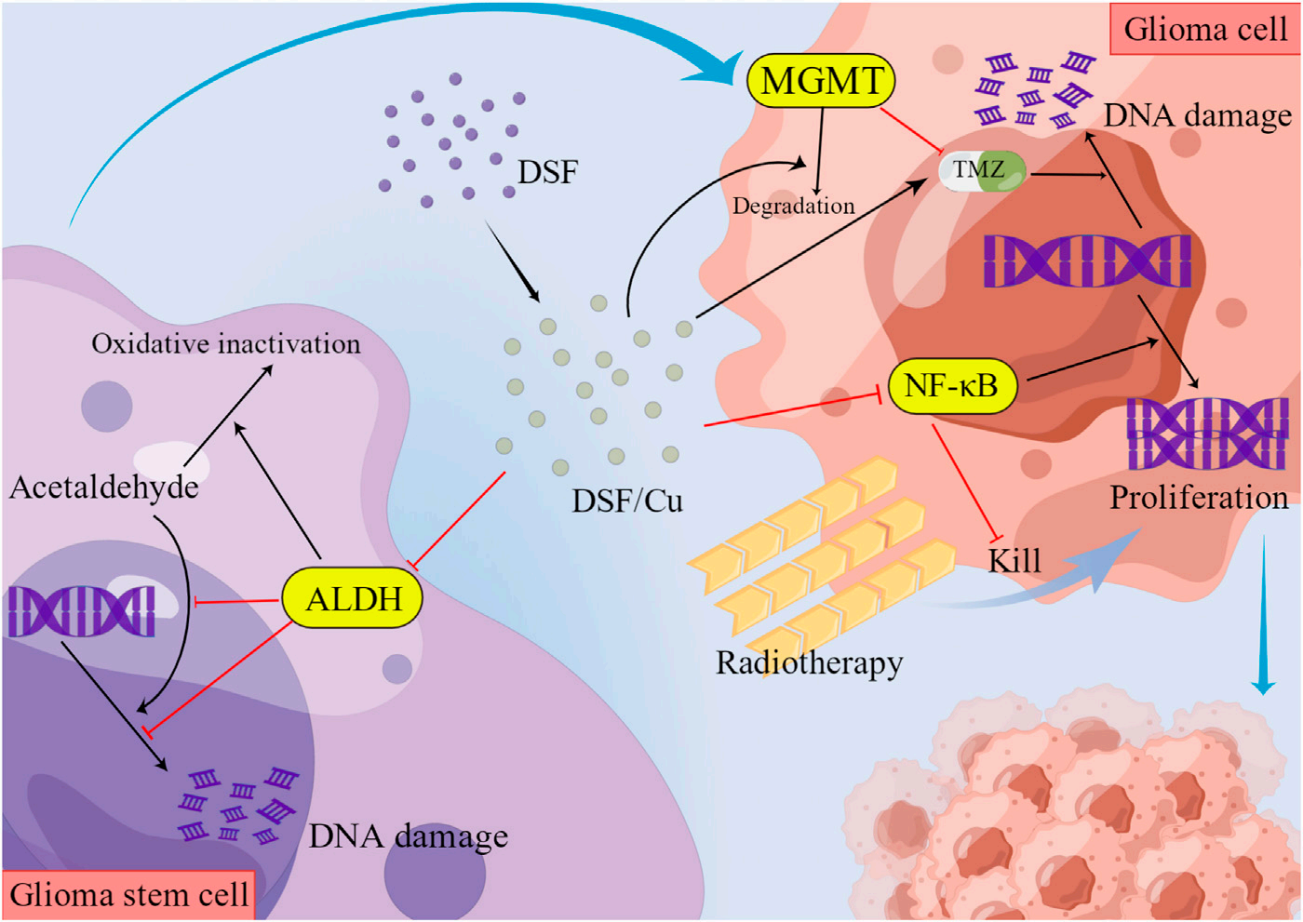
Molecular mechanisms of DSF.

DSF is involved in the regulation of complicated molecular mechanisms. These molecular mechanisms of DSF are shown in Figure 2 **.**

## 9 DSF delivery route

The standard dosage following FDA-approved indications for managing chronic alcohol use is 250 mg/day orally, with a maximum dose of 500 mg/day (Ekinci et al., 2019). Additionally, the nasal-brain pathway is considered a safe and effective alternative for direct drug delivery to the CNS (Landis et al., 2012). Administration via the nasal cavity to the CNS bypasses the blood-brain barrier and avoids hepatic first-pass effects (Qu et al., 2021). The clinical application of DSF as an anticancer drug is limited by its poor oral bioavailability and rapid metabolism *in vivo* (Shergill et al., 2016). It has been shown that DSF encapsulation in hydroxypropyl-β-cyclodextrin (HP-β-CD) produces a DSF complex with enhanced solubility (Qu et al., 2021). *In vitro* anti-GBM activity and safety (DSF/HP-β-CD/Cu) can increase the aqueous solubility of DSF by approximately 2,450-fold and may be a promising intranasal agent to treat (Qu et al., 2021). Animal studies have shown that DSF/HP-β-CD/Cu significantly inhibited tumor growth and migration, promoted tumor apoptosis, and prolonged the median survival time in male glioma rats in the intranasal administration group (Qu et al., 2021). Besides oral and nasal-brain access, 1,2-distearoyl-sn-glycero-3-phosphocholine (DSPC)/cholesterol liposomes to prepare DSF as an injectable Cu(DDC)_2_ formulation are also therapeutically active (Wehbe et al., 2017).

## 10 Combination therapy of disulfiram

Combinations of two or more drugs with different mechanisms of action, also referred to as combination therapy, is an alternative strategy for improving the success of drug repurposing (Masoudi-Sobhanzadeh et al., 2019). Compared with single-drug therapy, it reduces the incidence/emergence of resistance mechanisms, doses of drugs, and adverse effects, and improves the synergistic effects and success of treatment modalities (Masoudi-Sobhanzadeh et al., 2019). Combination therapy allows simultaneous targeting of multiple therapeutic genes, and is currently the most effective treatment for aggressive tumors, such as GBM (Ghosh et al., 2018). Combination therapy of DSF with other modalities holds great promise.

### 10.1 Disulfiram in combination with metal supplements

Preclinical and clinical studies have shown that DSF has broad-spectrum anticancer activity against a variety of cancer types when combined with Cu-containing supplements, such as Cu gluconate (McMahon et al., 2020). The combination regimen comprised 500 mg of DSF daily plus 50 mg of zinc gluconate or 2 mg of Cu gluconate three times daily (Ekinci et al., 2019). It has been shown that DSF/Cu sulphide (CuS) nanocomplexes (Tf-DSF/CuS) modified with transferrin (Tf) exhibit high cytotoxic effects *in vitro* (Lan et al., 2021). The CuS nanoparticles enable them to accumulate specifically and within tumor tissue by enhancing the permeability and retention (EPR) effect of the tumor tissue (Lan et al., 2021). The metallic nature of the nanoparticles enhanced the drug-loading capacity by forming Cu complexes on the CuS surface (Lan et al., 2021). Additionally, the use of a long-wavelength laser in the near-infrared (700–1000 nm) region activated the photothermal effect of the nanoparticles, resulting in minimal damage to normal tissue from tumor ablation (Lan et al., 2021). Besides Cu, DSF can act through zinc chelation to inhibit the activities of MMP-2 and MMP-9 (Kast and Halatsch, 2012).

### 10.2 Disulfiram in combination with standard chemotherapy drugs

*In vitro* cytotoxicity is enhanced by the addition of DSF to standard chemotherapeutic agents (Rappa et al., 2013) such as cisplatin (O'Brien et al., 2012), TMZ (Triscott et al., 2012), paclitaxel (Yip et al., 2011), gemcitabine (Cunningham and Chaiton, 2018), doxorubicin (Budman and Calabro, 2002), cyclophosphamide (Moreb et al., 2012), 5-fluorouracil (Wang et al., 2003), and adriamycin (Xu et al., 2011). The combination regimens for DSF against gliomas are described below.

#### 10.2.1 Disulfiram in combination with temozolomide

The 5-year survival rate of patients with GBM treated with radiation alone was 1.9%, and TMZ, in conjunction with radiation therapy, only increased the rate to 9.8% (Triscott et al., 2015). Additionally, TMZ toxicity is often not well tolerated by patients, and other modalities of TMZ resistance, such as MGMT expression, make it complicated (Triscott et al., 2015).

DSF is highly effective in cases where cells develop TMZ resistance (Triscott et al., 2012). DSF has synergistic activity when combined with TMZ, which is highly effective against TMZ-resistant cells (Lun et al., 2016). In the presence of low Cu doses, DSF significantly enhanced TMZ activity *in vitro* (Lun et al., 2016). *In vivo* studies have confirmed that DSF and Cu have synergistic effects with TMZ and improve survival in mice with *in situ* GBM tumors (Lun et al., 2016).

DSF can be safely used in combination with TMZ, although it causes reversible neurotoxicity (Huang J. et al., 2016). At a maximum tolerated dose (MTD) of 500 mg/day of DSF with TMZ without concomitant Cu administration, slight proteasomal inhibition of peripheral blood cells was observed after 4 weeks, with an average reduction of approximately 5% (Huang J. et al., 2016). A dose-response trend appeared with doubling proteasome inhibition at a dose of 1000 mg/day (Huang J. et al., 2016). The results of a phase II clinical study in 23 patients showed that adding DSF/Cu to TMZ was safe and well tolerated in TMZ-resistant IDH wild-type GBM (Huang et al., 2019).

#### 10.2.2 Disulfiram in combination with cisplatin

DSF enhances cisplatin-induced cytotoxicity by directly damaging DNA (O'Brien et al., 2012). Activating transcription factor 3 (ATF3) exerts a pro-apoptotic effect in response to cisplatin by directly binding to and activating vascular endothelial cells (O'Brien et al., 2012). It has been shown that the combination of cisplatin and DSF plays a synergistic role in inducing ATF3 protein expression and promoting tumor cell death (O'Brien et al., 2012). Adding DSF to the combination regimen of cisplatin and vincristine is well tolerated in antitumor therapy (Nechushtan et al., 2015). A dose of 40 mg DSF, with a half-life of approximately 7 hours, can be administered three times daily to improve its therapeutic effect with minimal side effects (Nechushtan et al., 2015). Significant neurotoxicity has not been reported with this combination therapy, besides fatigue (Nechushtan et al., 2015).

DSF may assist cisplatin in enhancing cytotoxic effects. This strategy appears to be safe, but the *in vivo* efficacy in glioma patients remains unclear.

#### 10.2.3 Disulfiram in combination with auranofin

A study of glioma stem cells in children found that DSF killed glioma stem cells at low concentrations and killed cell lines at slightly higher concentrations (Meier et al., 2021). The addition of auranofin increased DSF efficiency, and the synergistic effect was more pronounced in differentiated cells than in undifferentiated cells (Meier et al., 2021). Auranofin was also used in combination with DSF in the CUSP9 treatment program (Skaga et al., 2019).

#### 10.2.4 Combination with coordinated undermining of survival paths

DSF with aprepitant, auranofin, captopril, celecoxib, itraconazole, minocycline, quetiapine, and sertraline constituted a CUSP9 regimen that was used in combination with TMZ to synergistically disrupt the active survival pathway in GBM, block multiple signaling pathways, and make GBM vulnerable to the cytotoxic effects of TMZ (Skaga et al., 2019). A study of patient-derived glioblastoma stem cell (GSC) cultures from 15 patients with GBM showed that combining CUSP9 with TMZ produced a synergistic effect compared to the single drug (Skaga et al., 2019). CUSP9, combined with TMZ, was more effective than TMZ monotherapy in terms of the clinical plasma concentrations (Skaga et al., 2019). The CUSP9* regimen was generated based on the CUSP9 regimen, comprising DSF in combination with aprepitant, artesunate, auranofin, captopril, celecoxib, itraconazole, sertraline, and ritonavir (Kast et al., 2014). All nine drugs in the CUSP9* regimen were FDA-approved, each inhibiting one or more of the important GBM growth pathways (Kast et al., 2014).

DSF and auranofin have synergistic effect even without the other components of a CUSP9 regimen. All these might be the powerful adjuncts to TMZ chemotherapy. However, the side effects of CUSP9 or CUSP9* regimens combined with multiple agents deserve further clinical study.

#### 10.2.5 Disulfiram in combination with gemcitabine

Gemcitabine (2,2′-difluorodeoxycytidine, dFdC) is a deoxyribonucleic acid analogue that can be used as a single agent or combined with other anticancer drugs (Metro et al., 2010). Gemcitabine is active against a wide range of hematological and solid cancers. It is one of the few classical anticancer drugs that can pass through the BBB, penetrate the tumor mass, and be effectively converted to its active form in GBM tissue (Metro et al., 2010). However, its use in GBM chemotherapy is limited by the high resistance of GBM cells to dFdCs (Liu et al., 2012). Therefore, it is currently used mainly in combination with radiotherapy as a radiosensitizer for GBM treatment (Liu et al., 2012). DSF can synergistically enhance gemcitabine cytotoxicity and reverse gemcitabine resistance in cancer cell lines through ROS induction and inhibition of the ALDH and NF-κB pathways (Liu et al., 2012; O'Brien et al., 2012). In preclinical studies, DSF and its derivative pyrrolidine dithiocarbamate have been successfully used to enhance gemcitabine efficacy, particularly through NF-κB inhibition and oxidative stress generation (Iljin et al., 2009; Tesson et al., 2017). Increased oxidative stress due to DSF/Cu interactions with glutathione sensitizes cancer cells to gemcitabine treatment (Iljin et al., 2009; Tesson et al., 2017).

#### 10.2.6 Disulfiram in combination with carbenoxolone

Carbenoxolone is mainly used to treat gastric ulcers and other types of inflammation (Connors, 2012) and can play a role in inhibiting tumor cell growth by interfering with intracellular signaling through inhibiting ligand proteins (Zhang et al., 2003). DSF and carbenoxolone inhibit distinct interactions of GBM with the brain tissue microenvironment and stress-induced GBM cell-matrix adhesion with gap junction-mediated intercellular communication (Mettang et al., 2018). Animal experiments have shown that the combined use of DSF, carbenoxolone, and TMZ reduces tumor size in an *in situ* mouse model (Mettang et al., 2018). Tumor-initiating and adherent differentiated cells form gap junctions, and carbenoxolone can block adherent differentiated cells and affect intercellular communication (Mettang et al., 2018). Adherent differentiated cells are more sensitive to DSF treatment, and DSF interferes with cell-matrix adhesion by modulating NF-κB signaling (Mettang et al., 2018).

Table 2 shows the candidate chemicals used in combination with DSF for cancer treatment.

**TABLE 2:** The candidate chemicals used in combination with DSF for anticancer therapy.

Combination of drugs	Tumor type	Evidence type	Year and References
Temozolomide (TMZ)	Glioblastoma (GBM)	Clinical trials	Huang et al. (2019)
	Glioblastoma stem cells (GSCs)	Cells	Zirjacks et al. (2021)
Cisplatin	Ovarian *Cancer*	Animals	Bai et al. (2021)
	Head and neck squamous cell carcinoma (HNSCC)	Cells	Yao et al. (2021)
	Mammary cancer	Cells	Yang et al. (2019)
	Prostate adenocarcinoma	Cells	O'Brien et al. (2012)
	Atypical teratoid/rhabdoid tumor (AT/RT)	Animals	Jangra et al. (2020)
	Bladder cancer	Cells	Kita et al. (2019)
	Testicular germ cell tumors	Animals	Schmidtova et al. (2019)
	Esophageal squamous cell carcinoma	Animals	Jivan et al. (2018)
	Metastatic non-small cell lung cancer	Clinical trials	Nechushtan et al. (2015)
	Nasopharyngeal carcinoma (NPC)	Animals	Li et al. (2020)
Auranofin	Pediatric glioma	Cells	Meier et al. (2021)
	Hepatoma	Cells/Animals	Huang et al. (2016a)
	Ovarian cancer	Cells	Papaioannou et al. (2014)
Gemcitabine (dFdC)	Glioblastoma (GBM)	Cells	Tesson et al. (2017)
	Mammary cancer	Animals	Liu et al. (2021a)
	Pancreatic ductal adenocarcinoma (PDAC)	Cells/Animals	Kim et al. (2013)
	Colon cancer	Cells	Guo et al. (2010)
Regorafenib	Glioblastoma (GBM)	Animals	Zhao et al. (2018)
Carbenoxolone	Glioblastoma (GBM)	Animals	Mettang et al. (2018)
CUSP9/ Temozolomide (TMZ)	Glioblastoma stem cells (GSCs)	Cells	Skaga et al. (2019)
Paclitaxel	Mammary cancer	Cells	Liu et al. (2013)
	Lung adenocarcinoma	Animals	Mohammad et al. (2019)
Docetaxel	Mammary cancer	Cells	Swetha et al. (2020)
Doxorubicin	Osteosarcoma	Cells	Mandell et al. (2022)
	Mammary cancer	Cells	Rolle et al. (2020)
	Acute myeloid leukemia (AML)	Cells	Xu et al. (2011)
5-Fluorouracil (5-Fu)	Cervical carcinoma	Cells	Abidin et al. (2020)
	Pancreatic ductal Adenocarcinoma (PDAC)	Animals	Cong et al. (2017)
	Colorectal cancer (CRC)	Cells	Wang et al. (2003)
Arsenic trioxide (ATO)	Pancreatic cancer	Animals	Dinnen et al. (2013)

All references listed in the table are the latest research progress.

### 10.3 Disulfiram in combination with radiotherapy

DSF has a radiosensitizing effect on GBM cells (Koh et al., 2019) and enhances the radiosensitivity of AT/RT cell lines by increasing DNA damage, apoptosis, and autophagy (Lee et al., 2017). Combining DSF and Cu enhanced radiosensitivity by inducing cell death or interfering with DNA repair (Liu C. C. et al., 2021).

Thus, the efficacy of both chemotherapy and radiotherapy might be enhanced by DSF.

## 11 Adverse effects of disulfiram

DSF is a safe and well-tolerated drug, with mild side effects (Shirley et al., 2021). With chronic lymphocytic leukemia and normal lymphocytes (Wickström et al., 2007), invasive cancer and normal endothelial cells (Shian et al., 2003), and glioblastoma and normal astrocytes (Hothi et al., 2012), DSF is selectively toxic and kills human cancer cells (Paranjpe et al., 2014). The ability of different organs to resist endogenous and environmentally derived alkylating agents may be compromised, and this unrepaired DNA damage, particularly in regulatory oncogenes, may manifest as deleterious mutations and promote genomic instability (Paranjpe et al., 2014). DSF combined with Cu has an enhanced role in killing cancer cells; however, Cu-mediated cytotoxicity is also significantly increased in normal cells (Choi et al., 2015). The combined use of Cu and Zn in therapy is potentially dangerous because they are teratogenic and may lead to developmental defects (Choi et al., 2015). High DSF doses are hepatotoxic (Triscott et al., 2015), and rare cases of severe atopic hepatitis may occur, along with a risk of neuropathy; however, these symptoms are reversible after discontinuation (Huang J. et al., 2016). The mechanisms of neurological side effects of DSF remain unclear and may involve free acid radicals, inhibition of certain enzymes, calcium-induced neuronal toxicity, synergistic activity of neurotoxic drugs/chemicals, and other mechanisms (Kulkarni et al., 2013). If ethanol is ingested during DSF treatment, the large amounts of acetaldehyde produced from ethanol can cause severe nausea, headache, vomiting, flushing, and physical discomfort (Kast and Belda-Iniesta, 2009).

DSF is considered a relatively safe treatment since most of its adverse effects resolve after discontinuation. The mechanism of neuropathy caused by DSF still requires further research.

## 12 Conclusion

Malignant gliomas have a poor prognosis and high recurrence rate, posing a major threat to global public health. Current conventional treatment modalities hardly eradicate gliomas; therefore, new therapeutic approaches are urgently needed. Drug repurposing approaches have provided new research ideas for glioma treatment. This can help the pharmaceutical industry and researchers identify new uses for existing drugs. The expanding research on gliomas through drug repurposing approaches has made DSF a potential adjuvant for glioma treatment. DSF has a good safety profile and is an economical drug that is expected to play a broader role in the future treatment of gliomas. Further epidemiological studies should be performed to investigate the relationship between DSF and the risk to or survival of patients with gliomas. More clinical trials are needed to further refine treatment options for DSF. However, drug repurposing may not yet reach its full potential in the field of glioma, and new therapeutic agents through drug repurposing deserve further exploration.
